# Differential effects of TRPV1 receptor ligands against nicotine-induced depression-like behaviors

**DOI:** 10.1186/1471-2210-11-6

**Published:** 2011-07-18

**Authors:** Tamaki Hayase

**Affiliations:** 1 Department of Legal Medicine, Kyoto University, Kyoto 606-8501, Japan

## Abstract

**Background:**

The contributions of brain cannabinoid (CB) receptors, typically CB1 (CB type 1) receptors, to the behavioral effects of nicotine (NC) have been reported to involve brain transient receptor potential vanilloid 1 (TRPV1) receptors, and the activation of candidate endogenous TRPV1 ligands is expected to be therapeutically effective. In the present study, the effects of TRPV1 ligands with or without affinity for CB1 receptors were examined on NC-induced depression-like behavioral alterations in a mouse model in order to elucidate the "antidepressant-like" contributions of TRPV1 receptors against the NC-induced "depression" observed in various types of tobacco abuse.

**Results:**

Repeated subcutaneous NC treatments (NC group: 0.3 mg/kg, 4 days), like repeated immobilization stress (IM) (IM group: 10 min, 4 days), caused depression-like behavioral alterations in both the forced swimming (reduced swimming behaviors) and the tail suspension (increased immobility times) tests, at the 2 h time point after the last treatment. In both NC and IM groups, the TRPV1 agonists capsaicin (CP) and olvanil (OL) administered intraperitoneally provided significant antidepressant-like attenuation against these behavioral alterations, whereas the TRPV1 antagonist capsazepine (CZ) did not attenuate any depression-like behaviors. Furthermore, the endogenous TRPV1-agonistic CB1 agonists anandamide (AEA) and N-arachidonyldopamine (NADA) did not have any antidepressant-like effects. Nevertheless, a synthetic "hybrid" agonist of CB1 and TRPV1 receptors, arvanil (AR), caused significant antidepressant-like effects. The antidepressant-like effects of CP and OL were antagonized by the TRPV1 antagonist CZ. However, the antidepressant-like effects of AR were not antagonized by either CZ or the CB1 antagonist AM 251 (AM).

**Conclusions:**

The antidepressant-like effects of TRPV1 agonists shown in the present study suggest a characteristic involvement of TRPV1 receptors in NC-induced depression-like behaviors, similar to those caused by IM. The strong antidepressant-like effects of the potent TRPV1 plus CB1 agonist AR, which has been reported to cause part of its TRPV1-mimetic and cannabimimetic effects presumably via non-TRPV1 or non-CB1 mechanisms support a contribution from other sites of action which may play a therapeutically important role in the treatment of NC abuse.

## Background

Nicotine (NC) is the addictive substance in tobacco which results in increased use among adolescents and numerous harmful health effects have been reported for both males and females [1-3]. Its ability to alter the level of mood (e.g. depression, anxiety, etc.) is a characteristic of NC, as previously reviewed [4,5]. Depression is one of the most frequently-observed psychiatric symptoms associated with NC abuse, and has been reported mainly as a withdrawal symptom which occurs in dependent smokers [6,7]. Furthermore, in some daily smokers, direct depressant effects, which disappear soon after the cessation of smoking, have also been reported, and this is consistent with some animal experimental data [8-10]. On the other hand, in some clinical cases, brief antidepressant-like effects are observed during the period immediately after transdermal NC patch treatment [11]. The occurrence of both depressant and antidepressant effects seems to be one of the characteristics of NC-induced behavioral responses, and this occurrence of reduced depression has been postulated to reinforce the habitual use of NC, based on a review of clinical cases [12].

NC-induced "depression-like" behavioral alterations in animal experimental models have been quantified as exacerbated immobility in behavioral tests, such as the forced swimming test [9,10,13]. This test is used for screening antidepressants, which suppress immobility in swimming behaviors [14]. On the other hand, various stressors such as immobilization stress (IM) are known to cause depression-like behaviors, as represented by exacerbated immobility in swimming behaviors [15,16]. Repeated NC administration also caused exacerbated immobility in these behavioral tests [9,10,13].

In the author's preliminary studies, NC-induced depression-like behavioral alterations in mice were reduced by some antidepressants, which have been used to treat major depression and to antagonize brain nicotinic acetylcholine receptors (nAChRs), the direct targets of NC [10,17,18]. The effects of such antidepressants have recently been characterized by their ability to cause neurogenesis in the hippocampus [19,20]. Brain cannabinoid (CB) receptors, typically CB1 (CB type 1) receptors, can be considered one of the potent "antidepressant" targets of NC, based on their contribution to neurogenesis in the hippocampus via endogenous ligands [21,22] and the NC-altered levels of endogenous CB1 ligands in the brain including the hippocampus [23]. A direct contribution of CB1 receptors to some NC-induced alterations in locomotor activities was demonstrated by experiments using CB1 knockout mice [24]. In addition to CB1 receptors, recent immunohistochemical and behavioral studies have shown that transient receptor potential vanilloid 1 (TRPV1) receptors in the basal ganglia also interact with some endogenous CB1 ligands, and contribute to aberrant behaviors such as prolonged hypokinesia [25-28].

There have been few studies on the contribution of TRPV1 receptors to NC-induced behavioral effects, although antidepressant actions have been demonstrated for TRPV1 agonists such as olvanil [29]. Furthermore, the existence of a number of "candidate" endogenous TRPV1 ligands, which are expected to provide therapeutic effects against pathological conditions such as behavioral disorders, has been reported [30,31]. In the present study, based on the involvement of TRPV1 receptors in the effects of CB1 ligands, the functional relationship between nAChRs and TRPV1 receptors [32], and the author's preliminary data on the antidepressant-like effects of some cannabimimetics against NC-induced depression-like behaviors [10], the effects of TRPV1 ligands with or without affinity for CB1 receptors were examined against NC- and IM-induced depression-like behaviors in mice. Since few references on the depression-related behavioral alterations caused by TRPV1 ligands are available, the TRPV1 ligands were selected from those which provided some behavioral effects upon intraperitoneal or intravenous injections: the prototypic agonist capsaicin (CP) [33], the potent and antidepressant agonist olvanil (OL) [29], the antagonist capsazepine (CZ) [29], the endogenous TRPV1-agonistic CB1 agonists anandamide (arachidonylethanolamide: AEA) [27] and N-arachidonyldopamine (NADA) [34], as well as the synthetic TRPV1 and CB1 agonist arvanil (AR) [35].

## Methods

### Animals

Based on a previous study on stressor treatments [16], male ICR mice (80 ± 10 days old) (Shizuoka Laboratory Animal Center, Hamamatsu, Japan) were housed in a forced-air facility, which was maintained at 23°C and 50% relative humidity, with a 12 h/12 h light/dark cycle. The mice were kept separately in single transparent cages measuring 23.5 × 16.5 × 12 cm, and were allowed water and rodent chow ad libitum. The experiments described in this report were conducted in accordance with the "Guidelines for Animal Experiments" of the institution (updated in 2007) [36], which are based on the National Institutes of Health Guide for the Care and Use of Laboratory Animals, and any pain experienced by the mice was minimized. These guidelines were approved by the institutional ethics committee for animal experiments [36]. All of the observations and evaluations were performed by a trained observer who was blinded to the treatment conditions and was not informed of the treatment conditions in advance. Each experimental group contained 10 mice.

### Drug and stressor treatments

The protocols for the NC and stressor treatments were determined based on preliminary experiments and previous studies examining their behavioral effects [9,10,15,16]. With respect to the NC treatment, repeated subcutaneous (s.c.) doses of NC which caused the depression-like behaviors most effectively were selected: a single s.c. dose of 0.3 mg/kg was administered daily for 4 days [9,10]. NC (Nacalai Tesque, Inc., Kyoto, Japan) was supplied in free-base form at 95% purity, and was freshly dissolved in saline to a volume of 5 ml/kg immediately before each administration. With respect to the stressor, treatments using IM, which have been demonstrated to cause depression-like behavioral effects, were used based on previous studies in rodent depression models [16]. In the present experiments, repeated IM treatments in which the peak effects were almost equivalent to the NC treatments in preliminary experiments were selected: 10 min of IM, which was induced by placing the mouse in a narrow space (diameter about 12 cm) in a vinyl bag with some breathing holes, was given once per day for 4 days.

The TRPV1 ligands capsaicin (CP), olvanil (OL), capsazepine (CZ), anandamide (arachidonylethanolamide: AEA), N-arachidonyldopamine (NADA), and arvanil (AR) were purchased from Tocris Cookson Inc. (Ellisville, Missouri, USA). With respect to the doses of the TRPV1 ligands, the doses for the repeated administrations were selected based on previous studies and preliminary experiments [27,29,33-35]. For each drug, the data were collected and shown for those intraperitoneal (i.p.) doses which induced no toxic behavioral alterations by themselves at the present time point: 0.1, 1 and 2.5 mg/kg for CP, 0.1, 1 and 2.5 mg/kg for OL, 0.1, 1 and 5 mg/kg for CZ, 0.1, 1 and 10 mg/kg for AEA, 0.1, 1 and 10 mg/kg for NADA, and 0.1, 1 and 2.5 mg/kg for AR. The TRPV1 ligands were administered 60 min before each NC or IM treatment, based on previous studies and preliminary experiments [10,37]. Furthermore, in the experiments examining the role of TRPV1 and CB1 receptors, the TRPV1 antagonist CZ and the CB1 antagonist AM 251 (N-(Piperidin-1-yl)-5-(4-iodophenyl)-1-(2,4-dichlorophenyl)-4-methyl-1H-pyrazole-3-carboxamide: AM), purchased from Tocris Cookson Inc., were used in combination with the effective TRPV1 ligands, based on those doses causing behavioral effects [38,39].

Since the TRPV1 ligands are not soluble in water, they were initially dissolved in dimethylsulphoxide (DMSO) (Nacalai Tesque Inc.) to one-third of the total volume, and then diluted with distilled water to a volume of 5 ml/kg. Since AEA, NADA, and AR were provided in ethanol solutions (Tocris Cookson Inc.), the ethanol was evaporated immediately before use under nitrogen gas, and the residues were initially dissolved in DMSO to one-third of the total volume, and then diluted with distilled water to a volume of 5 ml/kg [40,41]. The other TRPV1 ligands were also dissolved or diluted in DMSO to one-third of the total volume, and then diluted with distilled water to a volume of 5 ml/kg. In the NC- and IM-only groups, a mixed vehicle solution of DMSO and distilled water at the same ratio as the TRPV1 ligand solutions was injected instead of the TRPV1 ligands, 60 min before each NC or IM treatment. In the TRPV1 ligand-only groups, the same volumes of saline vehicle were injected instead of the s.c. injection of NC. In the control group without any drug or stressor treatment (control group), a control vehicle solution of DMSO and distilled water at the same ratio as the TRPV1 ligand solutions was injected instead of the TRPV1 ligands, and then the same volume of saline vehicle was injected instead of the NC or IM treatment. The drug and stressor treatments and each experimental session were performed between 15 and 19 h of the light cycle.

### Forced swimming test

Based on previous studies [10,14,16], a glass cylinder apparatus 33 cm in height and 18 cm in diameter containing 14 cm of water at 21-23°C was used for the forced swimming test. Using the activity-measuring and recording system Supermex-CompACT AMS instrument (Muromachi Kikai Co. Ltd., Tokyo, Japan), for which an infrared sensor was placed over the cylinder at a distance of 20 cm from the water and the frequency of each mouse crossing the area under the sensor while swimming was measured as the number of counts, the "time until immobility", the time after when only modest swimming behaviors necessary to avoid drowning (<60 counts/min under the present conditions), as well as the "activity counts (per 10 min)" which reflected the amount of swimming behaviors during a 10 min experimental period, was monitored as a parameter of the test. Considering the time course of the behavioral alterations [10], the evaluations of these parameters were performed at the 2 h time point after the last NC or IM treatment.

### Tail suspension test

Based on previous studies [10,42], a cardboard cube apparatus of 35 cm per side was used for the tail suspension test. The front surface of the apparatus was open, and each mouse was suspended by fixing its tail in the center of the upper surface using a tail hanger and non-irritating adhesive tape. As a parameter for the test, the total duration of immobility ("total immobility time") during the 6 min experimental period was calculated. Considering the time course of the behavioral alterations [10], the evaluation of the parameter was performed at the 2 h time point after the last NC or IM treatment.

### Statistical analysis

The data obtained were subjected to a two-way analysis of variance (ANOVA) for the factors NC or IM treatment × treatment using each TRPV1 ligand [29]. With respect to the experiments examining the role of TRPV1 and CB1 receptors, the data obtained were subjected to a three-way ANOVA for the factors NC or IM treatment × treatment using each effective TRPV1 ligand × treatment using the TRPV1 or CB1 antagonist [39]. For pairwise comparisons, post-hoc Bonferroni tests were performed [10]. All of the comparisons were performed using statistical software packages ("Excel Statistics" from Social Survey Research Information Co. Ltd. Inc., Tokyo, Japan). P values less than 0.05 were considered to be statistically significant.

## Results

### Effects of TRPV1 ligands against NC- and IM-induced depression-like behavioral alterations in the forced swimming test

**Figure 1:**
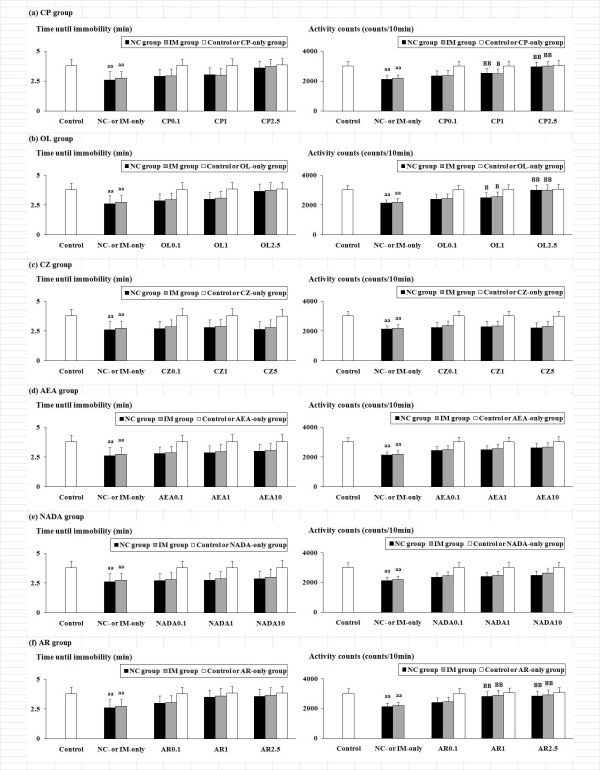
**NC- and IM-induced depression-related behavioral alterations and the effects of the six TRPV1 ligands in the forced swimming test**. The values for the time until immobility (min) and the activity counts (counts/10 min) at the 2 h time point after the last NC or IM treatment are shown. The data represent means ± SD (n = 10 for each group). aa (p < 0.01): significant attenuation as compared to the control group. B (p < 0.05), BB (p < 0.01): significant increase as compared to the NC- or IM-only group. The abbreviations of the co-administered TRPV1 ligands captioned below the X-axis with each i.p. dose (mg/kg) are noted in the text. The data for the control, NC-only, IM-only, and TRPV1 ligand-only groups are also shown.

The NC- and IM-induced depression-like behavioral alterations in the forced swimming test and the effects of the TRPV1 ligands are shown in Figure 1. In the NC- and IM-only groups, significantly attenuated "time until immobility" and attenuated "activity counts", i.e. the indices which reflected both the overall activity during the swim behaviors and the minimum activity after immobility, were observed as compared to the control group. In both NC and IM groups co-treated with CP (F(6, 108) = 3.46, P < 0.01), OL (F(6, 108) = 3.40, P < 0.01), or AR (F(6, 108) = 3.22, P < 0.01), the ANOVA with a 3 (NC, IM versus vehicle) × 4 (three doses of each TRPV1 ligand versus vehicle) factorial design revealed significant recoveries from the attenuated activity counts as compared to the NC- or IM-only group (Figure 1a, b, and 1f). On the other hand, in those NC and IM groups co-treated with CZ, AEA, or NADA, no significant interactions between the NC or IM treatment and the CZ, AEA, or NADA treatment were observed for either the times until immobility or the activity counts (Figure 1c, d, and 1e). However, each value was still significantly attenuated as compared to the control group (not shown in the figures). In each TRPV1 ligand-only group, no significant alterations for any parameters as compared to the control group were observed (white columns in Figure 1a-f).

### Effects of TRPV1 ligands against NC- and IM-induced depression-like behavioral alterations in the tail suspension test

**Figure 2:**
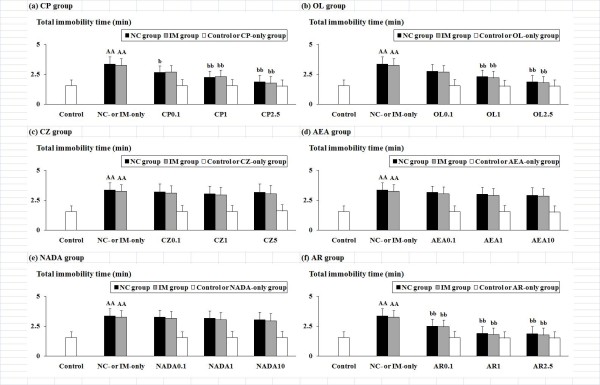
**NC- and IM-induced depression-related behavioral alterations and the effects of the six TRPV1 ligands in the tail suspension test**. The values for the total immobility time (min) at the 2 h time point after the last NC or IM treatment are shown. The data represent means ± SD (n = 10 for each group). AA (p < 0.01): significant increase as compared to the control group. b (p < 0.05), bb (p < 0.01): significant attenuation as compared to the NC- or IM-only group. The abbreviations of the co-administered TRPV1 ligands captioned below the X-axis with each i.p. dose (mg/kg) are noted in the text. The data for the control, NC-only, IM-only, and TRPV1 ligand-only groups are also shown.

The NC- and IM-induced depression-like behavioral alterations in the tail suspension test and the effects of the TRPV1 ligands are shown in Figure 2. In the NC- and IM-only groups, a significantly increased "total immobility time" as compared to the control group was observed. In both NC and IM groups co-treated with CP (F(6, 108) = 3.88, P < 0.01), OL (F(6, 108) = 3.70, P < 0.01), or AR (F(6, 108) = 4.21, P < 0.001), the ANOVA with a 3 (NC, IM versus vehicle) × 4 (three doses of each TRPV1 ligand versus vehicle) factorial design revealed significant recoveries from the increased total immobility times as compared to the NC- or IM-only group (Figure 2a, b, and 2f). On the other hand, in the NC and IM groups co-treated with CZ, AEA, or NADA, no significant interactions between the NC or IM treatment and the CZ, AEA, or NADA treatment were observed (Figure 2c, d, and 2e). However, each value was still significantly attenuated as compared to the control group (not shown in the figures). In each TRPV1 ligand-only group, no significant alterations as compared to the control group were observed (white columns in Figure 2a-f).

### Examination of the role of TRPV1 and CB1 receptors

**Figure 3:**
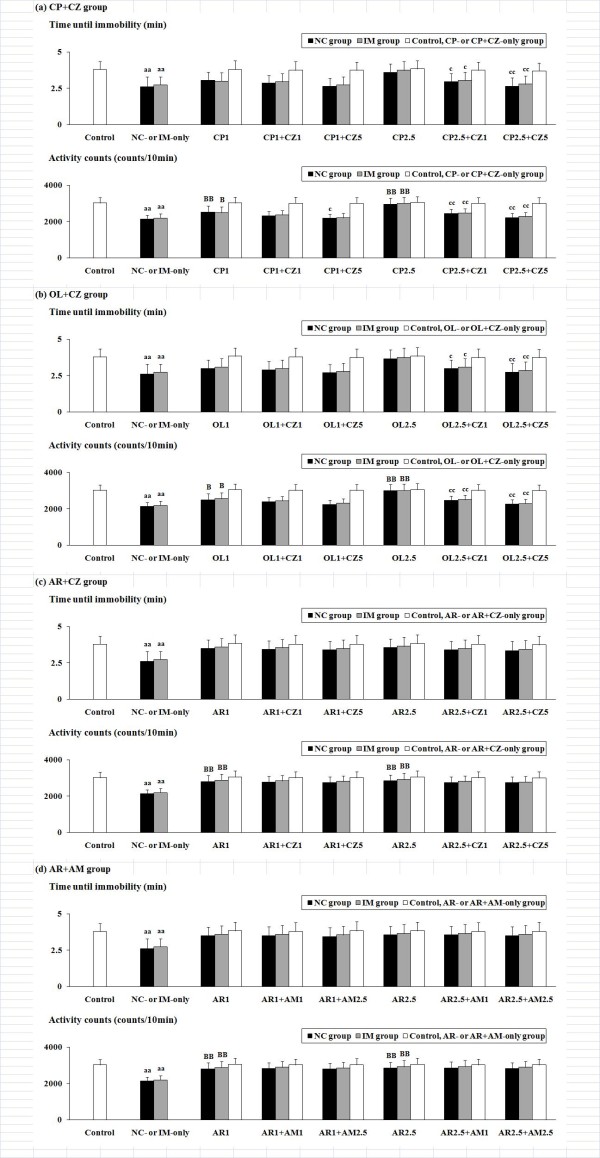
**Effects of the TRPV1 antagonist CZ or the CB1 antagonist AM against the depression-related behavioral alterations caused by NC or IM plus an efficacious (antidepressant-like) TRPV1 ligand (CP, OL or AR) in the forced swimming test**. Effects of CZ were examined in the TRPV1 agonist CP, OL or AR treatment group (a-c). Effects of AM were examined in the CB1 (plus TRPV1) agonist AR treatment group (d). The values for the time until immobility (min) and the activity counts (counts/10 min) at the 2 h time point after the last NC or IM treatment are shown. The data represent means ± SD (n = 10 for each group). aa (p < 0.01): significant attenuation as compared to the control group. B (p < 0.05), BB (p < 0.01): significant increase as compared to the NC- or IM-only group. c (p < 0.05), cc (p < 0.01): significant attenuation as compared to the NC or IM plus an efficacious TRPV1 ligand (CP, OL or AR) group. The abbreviations of the co-administered TRPV1 ligands with each antagonist captioned below the X-axis with each i.p. dose (mg/kg) are noted in the text.

**Figure 4:**
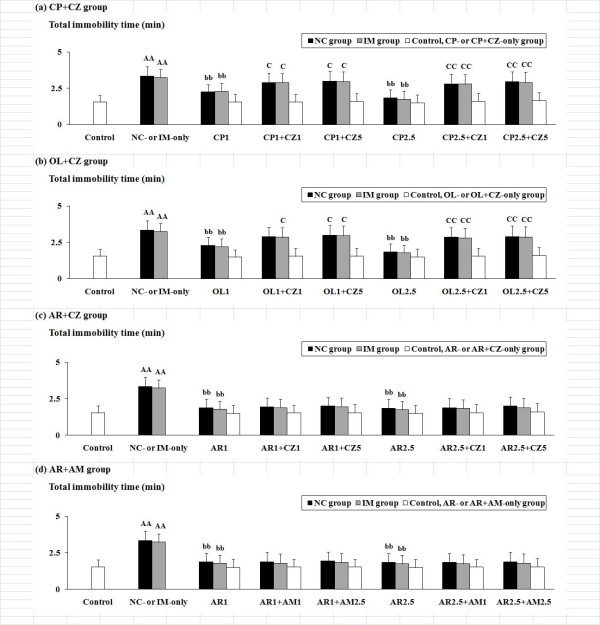
**Effects of the TRPV1 antagonist CZ or the CB1 antagonist AM against the depression-related behavioral alterations caused by NC or IM plus an efficacious (antidepressant-like) TRPV1 ligand (CP, OL or AR) in the tail suspension test**. Effects of CZ were examined in the TRPV1 agonist CP, OL or AR treatment group (a-c). Effects of AM were examined in the CB1 (and TRPV1) agonist AR treatment group (d). The values for the total immobility time (min) at the 2 h time point after the last NC or IM treatment are shown. The data represent means ± SD (n = 10 for each group). AA (p < 0.01): significant increase as compared to the control group. bb (p < 0.01): significant attenuation as compared to the NC- or IM-only group. C (p < 0.05), CC (p < 0.01): significant increase as compared to the NC or IM plus an efficacious TRPV1 ligand (CP, OL or AR) group. The abbreviations of the co-administered TRPV1 ligands with each antagonist captioned below the X-axis with each i.p. dose (mg/kg) are noted in the text.

With respect to the effective "antidepressant-like" drugs against the NC- and IM-induced behavioral alterations, interactions with the TRPV1 antagonist CZ (1 and 5 mg/kg) were examined for the TRPV1 agonists CP and OL, and interactions with both CZ (1 and 5 mg/kg) and the CB1 antagonist AM (1 and 2.5 mg/kg) were examined for the TRPV1 and CB1 agonist AR, in order to investigate the role of TRPV1 and CB1 receptors (Figure 3 and 4). In the CP and OL treatment groups, the ANOVA with a 3 (NC, IM versus vehicle) × 3 (two most effective doses of CP or OL versus vehicle) × 3 (two doses of CZ versus vehicle) factorial design revealed significant antagonistic interactions for CZ with the CP- or OL-induced antidepressant-like behavioral alterations in both the forced swimming and the tail suspension tests as follows: F(4, 243) = 2.89, P < 0.05 for the time until immobility (forced swimming test), F(4, 243) = 7.12, P < 0.001 for the activity counts (forced swimming test), and F(4, 243) = 5.28, P < 0.001 for the total immobility time (tail suspension test) in the CP treatment group; and F(4, 243) = 2.47, P < 0.05 for the time until immobility, F(4, 243) = 6.86, P < 0.001 for the activity counts, and F(4, 243) = 4.83, P < 0.001 for the total immobility time in the OL treatment group. In the AR group, however, no significant antagonistic interactions with the AR-induced antidepressant-like behavioral alterations were caused by either CZ or AM in the forced swimming or the tail suspension test.

## Discussion

### Similar "depression-like" behavioral alterations between NC and IM treatment groups

Consistent with a previous report [10], repeated NC doses under the present conditions caused enhanced "depression-like" behavioral alterations (i.e. reduced swimming behaviors in the forced swimming test and increased immobility time in the tail suspension test) as compared to the control group, similar to mice treated with a typical depression-inducing stressor [15]. However, as mentioned above, "antidepressant" effects for NC have also been reported clinically [11], and were also observed in some animal models [43-45]. In the present experimental model using ICR mice, short-lasting antidepressant-like behavioral alterations were also observed preliminarily at early time points (within 15 min) in the NC treatment groups. The occurrence of depression-like or antidepressant-like effects has been reported to be dependent on the specific animal strain and test [45]. It has also been predicted that a characteristically rapid desensitization of the direct target nAChRs, following their initial activation by NC, may yield contradicting depression-related behavioral alterations [45]. Furthermore, the NC-induced depression-related behavioral alterations may occur in accordance with neuroendocrine alterations such as the levels of brain monoamines and stress hormones, in which the time course coincides at least partly with the neuroendocrine responses caused by the depression-inducing stressors, regardless of the behavioral outcome [46,47].

As for the two behavioral tests used for the examination of depression-related behavioral alterations, it has been reported that the test batteries themselves cause different modifications in brain monoamine and plasma corticosterone levels depending on the test time [48,49]. However, under the present experimental conditions, the depression-related behavioral alterations were similar between the NC and IM groups, and were induced equally in both the forced swimming and the tail suspension tests.

### Effects of TRPV1 agonists vs. antagonists against NC-induced depression-like behaviors

Against the NC-induced depression-like behavioral alterations, as well as the IM-induced depression-like behaviors, a significant "antidepressant" attenuation was caused by the TRPV1 agonists CP and OL (Figure 1 and 2). Although the antidepressant-like effects of OL on the forced swimming behaviors are consistent with the data from a previous study [29], the antidepressant-like modifications of TRPV1 agonists against the effects of NC and IM have not been reported in representative behavioral tests. Furthermore, for these antidepressant-like effects, the involvement of TRPV1 receptors was demonstrated by their attenuation using the TRPV1 antagonist CZ (Figure 3 and 4), which has also been reported for the CP-induced hypolocomotor effects in the open-field test [33]. The author's data also strongly support the contribution of TRPV1 receptors to the NC- and IM-induced depression-like behavioral alterations.

The TRPV1 antagonist CZ by itself did not provide any significant effects against the NC- and IM-induced depression-like behavioral alterations. Considering that a blockade of TRPV1 receptors induced a reduced fear memory suggestive of an impairment in stress-coping [50] and anxiolytic-like behaviors via facilitating defensive responses [51], direct behavioral alterations caused by CZ could be predicted. However, in the present experimental model, no significant direct interactions were observed between CZ and the "depressogenic" NC and IM.

### Effects of cannabimimetic TRPV1 agonist against NC-induced depression-like behaviors

AR, a synthetic "hybrid" agonist of TRPV1 and CB1 receptors [35], also reduced the depression-like behaviors in both NC and IM groups for both tests. However, AEA and NADA, which function as both CB1 and TRPV1 agonists but less potently as TRPV1 agonists as compared to AR [26,27,52], did not evoke any antidepressant-like effects. In the author's preliminary study, even for lower doses as well as higher doses sufficient to cause toxic hypokinetic effects by i.p. injection, no significant behavioral effects were observed. On the other hand, previous studies have reported antidepressant-like effects following the intraperitoneal administration of the fatty-acid amide hydrolase inhibitor URB59 (3'-(aminocarbonyl)[1,1'-biphenyl]-3-yl-cyclohexylcarbamate) and the endocannabinoid transport inhibitor AM404 (N-(4-Hydroxyphenyl)-5Z,8Z,11Z,14Z-eicosatetraenamide), both of which enhance the levels of endogenous AEA [53,54]. Furthermore, a recent study showed antagonistic effects for AM404 against the NC-induced depression-like withdrawal symptoms via interactions between the endocannabinoid and serotonergic systems [55]. Although some direct CB1 agonists also provided antidepressant-like effects depending on the treatment conditions [54,56], it has been hypothesized that these drugs, unlike URB59 and AM404 which activate only target CB1 receptor subpopulations where endocannabinoids exert on-demand enhanced functions, activate whole-brain CB1 receptors including those subpopulations abolishing the antidepressant-related serotonergic responses [56]. Further studies are needed with respect to the effects of endogenous CB1 ligands, such as AEA and NADA, against NC- and IM-induced depression-like behaviors.

### "Antidepressant-like" effects of AR, a synthetic hybrid agonist for TRPV1 and CB1 receptors, against NC-induced depression-like behaviors

It has been reported that AR activates TRPV1 receptors with an efficacy at least comparable to that of CP [57]. Furthermore, it has also been reported that the potential of AR to activate brain CB1 receptors is at least comparable to that of AEA [57]. Nevertheless, in the present experimental model, the antidepressant-like effects of AR against the NC- and IM-induced depression-like behavioral alterations were not significantly blocked by the TRPV1 antagonist CZ or the CB1 antagonist AM 251 (Figure 3 and 4). In a previous study, some neurobehavioral alterations (e.g. catalepsy, attenuated spontaneous activity, hypothermia, analgesia) caused by systemic AR have been demonstrated to be unrelated to the activation of TRPV1 or CB1 receptors by experiments using TRPV1 and CB1 antagonists [35]. With respect to these effects of AR, the contributions of characteristic modifications in receptor-coupled responses such as glutamate release have been suggested [35,58]. However, the existence of non-TRPV1 and non-CB1 receptor-related mechanisms might be predictable, particularly against the complicated behavioral effects induced by NC or IM, although additional experiments including studies using TRPV1 and/or CB1 knockout mice are needed for the elucidation of the detailed mechanisms responsible for the effects of AR.

## Conclusions

TRPV1 receptors are known to participate in pain perception [59], and little is known about the TRPV1-related mechanisms underlying the depression-like behavioral alterations caused by NC similar to those caused by IM. Nevertheless, based on the results that the TRPV1 agonists CP and OL attenuated the NC-induced depression-like behavioral alterations, it is possible that TRPV1 receptors contribute, at least partially, to the NC-induced "depression". Further experimental studies are needed to confirm the additional neuronal mechanisms underlying the strong antidepressant-like effects of AR, which may be therapeutically important for the treatment of NC abuse.

## Authors' contributions

TH designed the study, carried out all experiments and statistical analyses, and prepared the manuscript.
